# Mid-Pleistocene Transitions Forced Himalayan ibex to Evolve Independently after Split into an Allopatric Refugium

**DOI:** 10.3390/biology12081097

**Published:** 2023-08-07

**Authors:** Gul Jabin, Bheem Dutt Joshi, Ming-Shan Wang, Tanoy Mukherjee, Stanzin Dolker, Sheng Wang, Kailash Chandra, Venkatraman Chinnadurai, Lalit Kumar Sharma, Mukesh Thakur

**Affiliations:** 1 Zoological Survey of India, Kolkata 700053, India; 2 Department of Zoology, University of Calcutta, Kolkata 700019, India; 3 Howard Hughes Medical Institute, University of California Santa Cruz, Santa Cruz, CA 95064, USA; 4 Kunming Institute of Zoology, Kunming 650223, China; 5 Zoological Survey of India, Marine Biology Regional Centre (MBRC), Chennai 600028, India

**Keywords:** allopatric speciation, *Capra sibirica*, demography, Himalayan ibex, taxonomic revision, Pleistocene glaciations

## Abstract

**Simple Summary:**

The Siberian ibex has a wide distribution, spanning various mountainous regions and cold deserts in countries like India, China, Pakistan, Kazakhstan, Tajikistan, Russia, and Mongolia. Our study specifically examined the Himalayan ibex, which is an edge population of Siberian ibex found in India. Through genomic analysis, we discovered that the Himalayan ibex evolved as a distinct lineage after separating from the main population around 100,000 years ago, due to geographical isolation. By studying the region’s past geography using paleo-climatic models and deep sequencing data, we observed significant transformations, including high mountains and deep valleys, particularly in the Pamir range, likely played a significant role in the divergence and independent evolution of the Himalayan ibex, after experiencing two historic genetic bottlenecks. Based on our findings, we propose prioritizing the Himalayan ibex as a unique phylogenetic species and advocate for its conservation attention and taxonomic revision. Furthermore, we hope that our research will inspire collaborative efforts and discussions among different countries to address the conservation and management of ibex populations worldwide.

**Abstract:**

Pleistocene glaciations had profound impact on the spatial distribution and genetic makeup of species in temperate ecosystems. While the glacial period trapped several species into glacial refugia and caused abrupt decline in large populations, the interglacial period facilitated population growth and range expansion leading to allopatric speciation. Here, we analyzed 40 genomes of four species of ibex and found that Himalayan ibex in the Pamir Mountains evolved independently after splitting from its main range about 0.1 mya following the Pleistocene species pump concept. Demographic trajectories showed Himalayan ibex experienced two historic bottlenecks, one each c. 0.8–0.5 mya and c. 50–30 kya, with an intermediate large population expansion c. 0.2–0.16 mya coinciding with Mid-Pleistocene Transitions. We substantiate with multi-dimensional evidence that Himalayan ibex is an evolutionary distinct phylogenetic species of Siberian ibex which need to be prioritized as *Capra himalayensis* for taxonomic revision and conservation planning at a regional and global scale.

## 1. Introduction

While species evolve through a process of millions of years of ecological and evolutionary successions, they also have a need for taxonomic recognition and planning for conservation [1,2]. Further, widespread species that are polytypic in nature are often neglected in policy planning [3,4,5]. However, a few recent studies advocated prioritizing peripheral populations of widespread species that evolved independently for regional management [6,7]. In this context, elucidating the impact of past geo-climatic events on widely distributed species would shed light on their genetic divergence and evolution [8,9]. Sporadic past geo-climatic oscillations, temporal topographic metamorphosis, and progressive changes in the mountainous system predominantly expedited species divergence by forming various allopatric refugia and their independent evolution following vicariant speciation [9,10]. Most mountain ungulates that evolved in temperate ecosystems experienced extreme geo-climatic transitions, which profoundly impacted their migration, colonization, refugial speciation, and extinction [11,12]. Using DNA-based approaches helps to understand the species’ population structure, taxonomy, and evolutionary history conditioned by the mentioned factors [13,14].

With this background, among the four well-recognized species in the genus *Capra*, i.e., Siberian ibex (*C. sibirica*), Alpine ibex (*C. ibex*), Nubian ibex (*C. nubiana*), and Iberian ibex (*C. pyrenaica*), Siberian ibex is one of the widely distributed ibex across the arid zone of central Asia [15,16]. Siberian ibex have its reported distribution in rugged terrains, highland flats, mountain ranges, and cold desert habitats of India, China, Pakistan, Kazakhstan, Tajikistan, Russia, and Mongolia [15,16]. Based on their horn size and body coloration, Fedosenko and Blank (2001) [15] characterized four sub-species in Siberian ibex, i.e., *C. s. hagenbecki* (Gobi desert), *C. s. sibirica*, (Altai mountains), *C. s. alaiana* (Tian Shan range), and *C. s. sakeen* (Pamirs, Hindu Kush, and Karakorum). Later on, Smith and Xie (2009) [17] added another sub-species, *C. s. dementievi*, from the Kunlun mountains, but Wilson and Reeder (2005) [18] questioned the existence of all these sub-species. In India, the natural populations of *C. sibirica* (*henceforth*; Himalayan ibex) are reported only from the Trans-Himalayan region of Ladakh and Himachal Pradesh [16]. It is listed as ‘*Near Threatened*’ under the International Union for Conservation of Nature (IUCN) Red List [16] and categorized under Schedule I of the Wild Life (Protection) Act, 1972 of India. Joshi et al. (2020) explained the preliminary patterns of genetic divergence of Himalayan ibex in India using a mitochondrial marker [19]. However, the sole use of mitochondrial DNA to interpret evolutionary patterns is often considered inadequate due to the stochasticity of genetic drift and incomplete lineage sorting [20,21]. Therefore, whole genome sequencing is preferred to validate evolutionary patterns, demographic history, and resolving taxonomic uncertainties [9,22,23,24]. In the present study, we hypothesized whether species divergence in Siberian ibex is facilitated by the Pleistocene species pump concept (PSPC). The Early to Middle Pleistocene Transition (c. 1.2–0.5 mya) represents a major episode in earth’s history that also brought fundamental changes in earth’s climatic cycle [25,26]. In this context, we analyzed 40 genomes from four species of ibex to test our hypothesis, where we assume the PSPC is pertinent to allopatric speciation in ibex, and the divergence time/the large demographic changes in Himalayan ibex match chronologically with the Middle Pleistocene Transitions.

## 2. Materials and Methods

### 2.1. Genome Sequencing, Variant Calling, and Phylogeny Based on Nuclear Variants

We collected three Himalayan ibex samples, one from Lahaul, Himachal Pradesh, and two samples from Ladakh, Union Territory, during 2019–2020, and extracted genomic DNA using Qiagen DNeasy Blood and Tissue Kit (Qiagen, Hilden, Germany). We prepared two paired-end libraries following Illumina TruSeq PCR-free HT library Prep Kit (Illumina, Inc., San Diego, CA, United States) and sequenced them on Illumina HiSeq 2500 platform. We screened raw reads for quality check using FastQC (http://www.bioinformatics.babraham.ac.uk/projects/fastqc/) (accessed on 13 September 2021), and filtered out short (<50 bp) and low quality reads (Q < 20) using trimmomatic-0.39.jar (“PE-threads 5-phred33 LEADING:3 TRAILING:3 SLIDINGWINDOW: 4:15 MINLEN:36”). The qualified reads were then aligned with the goat reference genome (GenBank: GU295658.1), using the BWA-MEM (Li 2014) with default settings “-t 4-M”. The resulted bam alignment files were processed, including coordinate sorting, duplicated reads marking, local realignment, and base quality recalibration using corresponding tools in Picard (version 1.56; http://picard.sourceforge.net (accessed on 14 september 2021)) and GATK-v3.7.0 [27]. We then mined 37 genomes, i.e., 21 Alpine ibex (*Capra ibex*), 7 Siberian ibex (*Capra sibirica*), 5 Iberian ibex (*Capra pyrenaica*), 3 Nubian ibex (*Capra nubiana*), and 1 of bezoar (*Capra aegagrus*) (Table S1). We called nuclear variants (SNPs) using the Unified Genotyper function in GATK-v3.7.0 and SNPs were filtered as described in [28] using the Variant Filtration command in GATK-v3.7.0 with parameters “QUAL < 40.0 MQ < 25.0 MQ0 ≥ 4 & ((MQ0/(1.0 × DP)) > 0.1) cluster 3 -window 10”. Index, depth, and mapping statistics were computed using SAMtools software [29]. We used a custom-made python script to convert ‘.vcf’ genotype for each sample into ‘.fas’ format. The control ‘.mao’ file was generated using MEGA-Proto phylogeny construction and NJ tree was constructed using mega-cc tool in MEGA X [30] following 1000 bootstraps.

### 2.2. Assembling Mitogenomes and Phylogeny

We extracted 40 mitochondrial genomes including three Himalayan ibex using ANGSD version: 0.933 [31] with settings “angsd -doFasta 1 -doCounts 1 -setMinDepth 6 -minMapQ 25 -minQ 25 -uniqueOnly 1 -nThreads 2”. We aligned mitogenome sequences using MUSCLE v3.8.31 [32] and the alignment file of 16,643 bp was used for phylogenic analysis after removal of poorly aligned segments. The NJ phylogenetic tree was reconstructed using RAxML v8.2.12 [33] following the GTR + G model of nucleotide substitution with 1000 bootstraps support. In addition, we also analyzed 66 sequences of mt cyt *b* gene (Table S2), i.e., 53 sequences of *C. sibirica* from various ranges (including 23 sequences of Himalayan ibex generated in the present study), 4 sequences of *C. nubiana*, 6 from *C. ibex*, and 2 sequences from *C. pyrenaica* with *Gazella gazelle* as an outgroup for phylogenetic analysis following Joshi et al. [19] (Table S2).

### 2.3. Population Genetic Assignment, Admixture, and Gene Flow

We scrutinized genomes of Siberian ibex for population genetics assignment using explicit non-Bayesian and Bayesian clustering algorithms with the Himalayan ibex genotypes. In the non-Bayesian method, we performed PCA in GCTA software Eigensoft package v 5.0.2 [34] and in the Bayesian method, we inferred population genetic structure using the program ADMIXTURE v 1.23 [35] by assuming that the number of ancestral populations (K) increased gradually. We pruned genotypes with parameters “—indep-pairwise 50 10 0.1” for both PCA and admixture clustering based on linkage disequilibrium by PLINK [36].

### 2.4. Demographic Estimations and Population Divergence

We scrutinized genomes with coverage >20-folds to ensure the accurate calling of heterozygotes and inferred historical demography using pair-wise sequential Markovian coalescent (PSMC) [37]. We generated a diploid consensus sequence for each individual using the “mpileup” command of the samtools package v 1.3.1 [29] with the option “-C50”. Variants with less than about 1/3 (“-d” option) or over 2 times (“-D” option) of average read depth were marked missing and excluded from consensus sequence assignment. We also filtered out sequences with low quality (Q < 20) and converted consensus sequences into 100-bp bin-input files using program “fq2psmcfa” in the PSMC package. We ran PSMC with parameters “−N25 − t15 − r5 − p 4 + 25 × 2 + 4 + 6” and PSMC estimates were scaled using a generation time (g) of 8 years and a mutation rate (µ) of 1.728 × 10^−8^ substitutions per site per generation following Brambilia et al. [38] and Chen et al. [39]. We also estimated divergence time between Himalayan ibex and other populations of Siberian ibex from different ranges and with the other ibex species using the multiple sequential Markovian coalescent (MSMC2) model [40]. Genotypes for all ibex were phased together using Beagle V.4.1 [41]. We used time as the relative cross coalescent rate (RCCR) which dropped to 50% and was used as a rough estimate of the splitting time [40,42]. For each model, we performed 100 independent runs with varying starting points. Similar to the PSMC analysis, we restricted this analysis to genomes with coverage >20-folds and followed a generation time (g) of 8 years and a mutation rate (µ) of 1.728 × 10^−8^ substitutions per site per generation following Brambilla et al., 2014 [38] and Chen et al., 2019 [39].

### 2.5. Genetic Affinity and Gene Flow

We explored genetic affinities and gene flow between Himalayan ibex and other populations of Siberian ibex from different ranges using TreeMix v1.13 [43] and ABBA-BABA test (also called D-statistics) [44]. Based on NJ tree clusters, we divided the genomes into six groups—Group MT (Himalayan ibex), Group 2 (*C. sibirica*–TJ/KZ and China I), Group 3 (*C. sibirica*–China II), Group 4 (*C. nubiana*), Group 5 (*C. pyrenaica*), and Group 6 (*C. ibex*). We constructed a maximum-likelihood (ML) tree by allowing gene flow in TreeMix v1.13 with all variants located on autosomes using 1000 variants per block (“-k 1000”) by allowing 0 to 6 migrations, respectively, with bezoar as outgroup. The ABBA-BABA test or D-statistics, an estimate of gene flow, is based on the topology (((H1, H2), H3), outgroup), where D = 0 refers to no gene flow between ingroup (H1 or H2) and H3; D > 0 refers to gene flow between H3 and H2; and D < 0 refers to gene flow between H3 and H1 [44]. We used the function “-doAbbababa 1” in ANGSD v0.931 to perform this analysis with the additional settings “-doCounts 1 -minMapQ 25 -minQ 25 -uniqueOnly 1 -nThreads 6”.

### 2.6. Modeling Geo-Climatic Changes

We deciphered changes in topography and climatic envelope across the distribution of Siberian ibex from India to Kazakhstan between present time and before the onset of Pleistocene glaciation, i.e., c. 2.8–3.0 mya. We prepared paleo digital elevation model (paleoDEM) at 1° following PaleoDEM Resource [45] and also accounted for terrain ruggedness index (TRI) to investigate the amount of elevation heterogeneity between the adjacent DEM pixels using GradientMetrics Tool Box [46]. Further, we used frequency distribution with natural bins for categorization and measurement of lower and higher elevation profiles between present and paleo DEM. For the present time, we also extracted annual mean temperature and precipitation data from the WorldClim database (https://worldclim.org/data/index.html (accessed on 22 September 2021)), using path tracking points (*n* = 13,814 geo-points). We selected geo-points distributed across all contours within the distribution of Siberian ibex to represent all possible topographic surface configurations.

## 3. Results

### 3.1. Phylogeny and Population Genetic Assignment of Himalayan ibex

**Figure 1:**
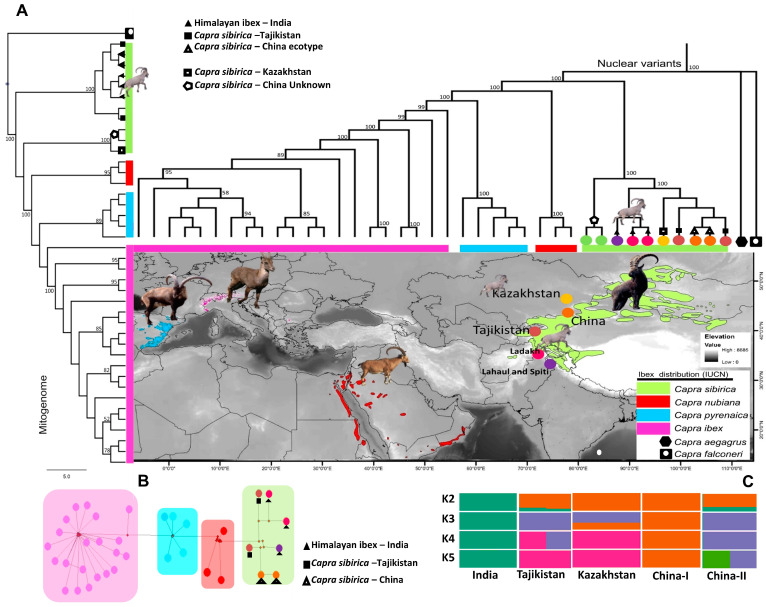
Phylogenetic relationship and population genetic assignment of Himalayan ibex with other populations of Siberian ibex (**A**) Phylogenetic relationship of Himalayan ibex in the genus *Capra* constructed based on autosomal variants (top of the map) and mitochondrial genome (left side of the map) following neighbor-joining method, where bezoar is used as an outgroup. Numbers on branches are bootstrap values (%) derived from 1000 replications. (**B**) Haplotype network tree of different species of ibex based on the complete mitochondrial genome. (**C**) ADMIXTURE results based on the 53 million SNPs mapped against goat reference genome. Population membership assignment is shown from K2 to K5.

We sequenced three ibex samples from India on 5.60, 5.63, and 29.66 coverage and mapped about 53 million SNPs with the goat reference genome (GenBank: GU295658.1). The Neighbor-Joining (NJ) trees based on the nuclear as well as mitochondrial genomes concurrently displayed four major clades in accordance with the four species of ibex, and the Himalayan ibex formed a paraphyletic clade within the clade of *C. sibirica* (Figure 1A). The mitogenomes-based haplotype network was also in agreement with the clustering patterns observed in the phylogenetic trees (Figure 1B).

The phylogeographic assignment of the Himalayan ibex among the Siberian ibex from various ranges (including the Altai mountains and Russia, for which no genome was available) based on the complementary sequences of mitochondrial cytochrome *b* gene formed four major clades in agreement with SNPs and mt genome-based phylogenies (Figures S1 and S2). In the population structure assignment using ADMIXTURE, we observed Himalayan ibex to be an isolated population at K 2 to 5 (Figure 1C) showing different signatures. In PCA, we observed at least five sub-clusters within Siberian ibex, i.e., one each from India, Tajikistan, and Kazakhstan, while samples from China formed two sub-groups (Figure S3).

### 3.2. Demographic History, Genetic Divergence, and Gene Flow

**Figure 2:**
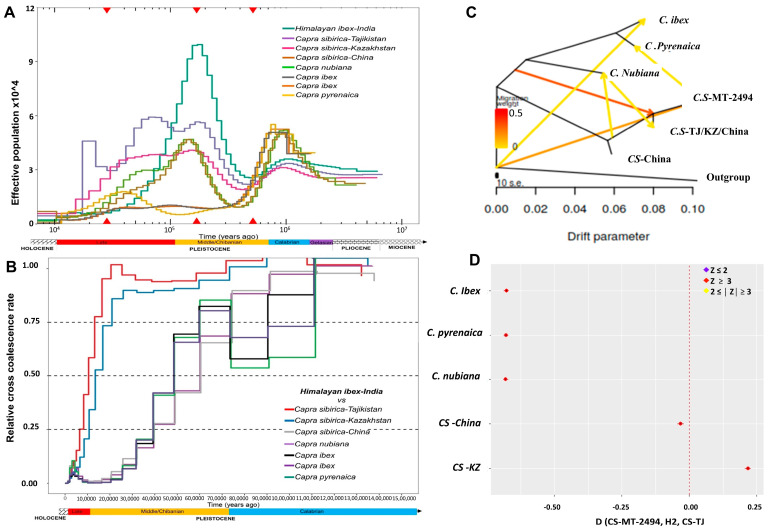
Gene flow, demographic history, and genetic divergence of Himalayan ibex with core population of Siberian ibex from various ranges and with other ibex species (**A**) PSMC result showing the demographic trajectories of Ibex. (**B**) MSMC result showing the split of Indian ibex from the other species and main population of *Capra Sibirica.* (**C**) TreeMix tree graph constructed by allowing six migration edges (m = 6). (**D**) D-statistics suggest complex admixture among different species and Himalayan ibex shared allele with Tajikistan and China compared to Kazakhstan.

The PSMC result in Himalayan ibex displayed a demographic trajectory different from other ibex populations. Himalayan ibex experienced two historic bottlenecks: one each between 0.8 and 0.5 mya and 50–30 kya with an intermediate large population expansion between 0.2 and 0.16 mya (Figure 2A). Tajikistan and Kazakhstan populations experienced historic events almost on the similar geological time scale. The first bottleneck event experienced by them was around c. 0.5–0.4 mya; then, both populations eventually expanded between c. 0.4 and 0.2 mya. The Kazakhstan population showed steady growth during 0.2–0.04 mya, while the Tajikistan population slightly increased during 0.1–0.060 mya. Both populations then declined abruptly with the onset of the LGP. The ibex population in China showed a population decline and experienced a bottleneck around 0.65 mya with a slight expansion around 0.5 mya; it further declined to experience another bottleneck around 0.3 mya (Figure 2A,B). Himalayan ibex show divergence from the Siberian ibex main population around 0.1 mya following allopatric speciation (Figure 2B).

The genetic affinity and gene flow analysis allowing 0–6 migration tree models unequivocally supported the phylogenetic results by confirming Himalayan ibex (MT group) as a paraphyletic clade to group 2 (Tajikistan, Kazakhstan, and Chinese populations of *C. sibirica*) (Figure 2C and Figure S4). D-statistics analysis [44] placed Himalayan ibex within the Siberian ibex clusters (Tables S3 and S4) and reveal that Himalayan ibex shared an excess of the allele with Tajikistan and Chinese ibex compared with the Kazakhstan population (Figure 2D and Table S3).

### 3.3. Paleo-Geo Climatic Modeling

**Figure 3:**
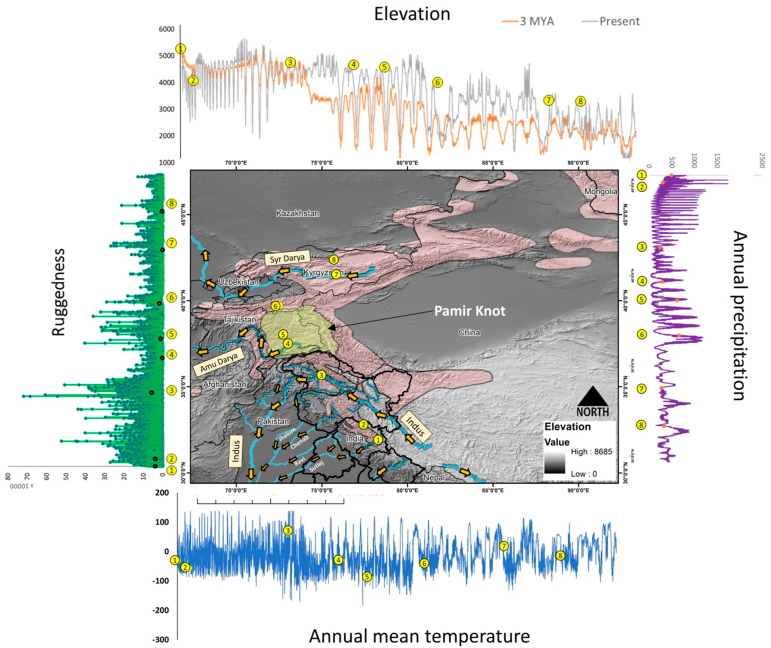
Illustration of topographic and climatic envelope of the study region. Yellow number represent values of different topographic and climatic variables of particular location corresponding to the location represented in the map.

In geo-climatic modeling, we observed substantial variation in both the lower and higher elevation profiles between paleoDEM and present elevation models (Figure S5; Table S4). We found deep valleys with a lower elevation gradient as well as mountain peaks with a high elevation gradient upraised over time (Table S5). Furthermore, we observed that valleys with lower elevation gradients became at least three times deeper from 88.26 km (c. 3 mya) to 260.16 km (present time) on the likely region of disjunction in the Pamir Mountains (geo-point #6) (Figure 3 and Table S6).

The fluctuation in the elevation profile is represented by the gray line (present) and by the orange line (3 mya) on the top of the figure. The variation in the ruggedness index within the landscape is represented by the green line on the left of the figure. The annual mean temperature is represented by the blue line on the bottom of the figure and annual precipitation is represented by the violet line on right of the figure.

## 4. Discussion

### 4.1. Phylogeny and Population Genetic Assignment of Himalayan ibex

The NJ trees indicate Himalayan ibex as an evolutionarily diverged and sister lineage within Siberian ibex. The mitogenomes-based haplotype network was also consistent with the phylogenetic trees and showed relatively more phylogenetic affinities with the Tajikistan population following the geographical proximities of these populations. In addition, based on the complementary sequences of the mitochondrial cytochrome *b* gene, we observed three clades with respect to the geographical origin of the samples: the I–T clade, representing India and Tajikistan; the KZ clade, representing Kazakhstan; and the AMR clade, representing sequences from the Altai Mountains, Mongolia, and Russia. Most interestingly, the I–T clade was found paraphyletic to the KZ and AMR clades which showed its adequate genetic divergence from other ranges. These analyses suggested Himalayan ibex to be an evolutionarily distinct lineage of Siberian ibex.

In the population structure assignment, Himalayan ibex are displayed as an isolated population at K 2 to 5 (Figure 1C), suggesting a unique genetic makeup of Himalayan ibex from other populations of Siberian ibex. This observation supports the assumption that Himalayan ibex did not share any gene pool with its adjacent populations in the recent past and remained geographically isolated after divergence. However, Tajikistan and Kazakhstan populations shared genetic ancestry suggesting possible gene flow during the recent past (Figure 1C). PCA also supports the former results, and that Himalayan ibex formed a distinct cluster, indicating its unique genetic makeup among other populations of Siberian ibex.

### 4.2. Demographic History, Genetic Divergence, and Gene Flow

Using PSMC, we could trace the demographic history of ibex populations up to 5 mya and, interestingly, we observed Himalayan ibex displayed a demographic trajectory different from other ibex populations. The Himalayan ibex early bottleneck coincided with the geological time scale of Middle Pleistocene Transitions (c. 1.25–0.7 mya) [47,48] that facilitated severe geo-climatic changes, and so caused abrupt decline in the large population of Siberian ibex. This period also marks the expansion of ice sheets severely impacting large groups of animals and plants [49,50] and with no exception the Siberian ibex severely declined and its peripheral population in the Pamir region were possibly trapped as a glacial refugium in the Indian Trans-Himalayan region (ITHR). Since the ice sheets melted with the onset of the interglacial period (MIS-15 and MIS-9) [51], the refugial population in ITHR would have started to expand in size c. 0.4 mya to reach a climax c. 0.18 mya (Figure 2A). Further, Penultimate Glaciations during c. 0.25–0.13 mya [52] would have also accelerated geographical disruption and wide separation of the Himalayan ibex on the southern side of the Pamir Mountains. Interestingly, historical events like the first population bottleneck (c. 0.8–0.5 mya), followed by population expansion (c. 0.2–0.16 mya) with simultaneous disjunction from its neighborhood population following allopatric speciation (c. 0.1 mya) as resulted by the MSMC analysis, all matched in chronological order with the geological time scale (Figure 2A,B). We postulate that Himalayan ibex would have been trapped as a glacial refugium during c. 0.5 mya after declining abruptly due to the extreme geo-climatic conditions during the Middle Pleistocene Transitions and eventually evolved independently without forming post-glacial secondary contact zones. These explanations are reasonable as we did not obtain any genetic admixing of Himalayan ibex with the neighboring Tajikistan population in the admixture analysis (Figure 1C). Further, the last bottleneck event appeared c. 50 to 30 kya, which coincided with the onset of the Last Glacial Period (LGP; 125–10 kya) [53,54] and with the local Chandra glaciations in ITHR (c. 40 and 78 kya) [55]. After the Last Glacial Maxima (LGM; c. 25–30 kyr) to the recent mid-Holocene epoch (c. 5 kyr), the continuous ice sheets melted and deep glacier valleys formed in the Himalayan region [49,55,56] and these transitions plausibly eroded the suitable habitats of Himalayan ibex to restrict them in the high altitude of the Trans-Himalayan ranges. Interestingly, the observed demographic patterns of Himalayan ibex and other ibex species during the past geo-climatic events were consistent with the species distributed in the adjoining region of the Qinghai Tibetan Plateau (QTP) [57,58]. Further, during the late Holocene, human settlements and their activities proliferated in the ITHR which also resulted in the further decline of Himalayan ibex until the present time [59,60]. Although population sizes considerably varied between Tajikistan and Kazakhstan populations, they experienced historic events almost on the similar geological time scale. The first bottleneck event experienced by them coincided with the glaciations dated c. 0.3–0.12 mya in the Kazakhstan Pamir region defined by Q2 morain [61]. Then, both populations eventually expanded between c. 0.4 and 0.2 mya coinciding with MIS-5a, the interglacial period [51]. The Kazakhstan population showed steady growth between 0.2 and 0.04 mya, while the Tajikistan population slightly increased between 0.1 and 0.060 mya. Both populations then declined abruptly with the onset of the LGP. The ibex population in China showed population decline to experience a bottleneck around 0.65 mya, which coincided with the Naynayxungla Glaciation (0.78–0.5 Ma) [52,62]. With a slight expansion around 0.5 mya, it further declined to experience another bottleneck around 0.3 mya, which likely caused by the Penultimate Glaciations (0.25–0.13 Ma) [52,62]. Then, the population underwent expansion around 80 kya which coincided with MIS-5a, the interglacial period [51] with the recent decline due to the arrival of the LGM (Figure 2A).

Our genetic affinity and gene flow analysis suggested complex admixture among *C. sibirica, C. ibex, C. pyrenaica*, and *C. nubiana* (Figure S4). We propose that evolution of ibex is not genetically isolated but a complex phenomenon which is consistent with the patterns observed in other species including Red jungle fowl [63,64], canids [22,65,66], *Bos* species [67], and felidae [68]. The excess of allele sharing using D-statistics analysis reveals that Himalayan ibex shared an excess of the allele with Tajikistan and Chinese ibex; however, sharing of alleles did not reflect a recent gene flow but possibly explain the shared ancestry of Himalayan ibex with Siberian ibex in the past.

### 4.3. Paleo-Geo Climatic Modeling

The lower and higher elevation profiles between paleoDEM and the present elevation models, in particular, the north and south of the Pamir Mountains, that support the contiguous distribution of Siberian ibex from India to Tajikistan experienced a significant topographic metamorphosis during the Pleistocene Transitions. The current patterns of ruggedness in the landscape corroborated with the present DEM, and suggested that ibex distribution from Lahaul, India, to Tajikistan (geo-points #1 to 6) were sustained with enormous undulation and substantial increase in the contours than relatively less heterogeneity in the landscape from Tajikistan to Kazakhstan (geo-points from #6 to 8) during the Pleistocene Transitions (Figure 3; ruggedness graph). Further, the annual mean temperature and precipitation, which are the major contributors of the climatic envelope supported by the mountains that lie in the south of the Pamir range to the border of Hindu Kush Mountains along Afghanistan’s Wakhan Corridor, have been warmer than the north of the Pamir range as they connect to Tian Shan mountains along the Alay Valley of Kyrgyzstan [69]. Since the annual temperature and precipitation regulate vegetation growth [48,49,63], we believe that after the geographical split of Himalayan ibex into glacial refugium during the Middle Pleistocene Transitions, this population would have experienced relatively more conducive ecological conditions than its counterparts in the north of the Pamir range. This plausibly facilitated Himalayan ibex to progressively expand, as considerable differences were observed in the population size and demographic history between populations confined to the north (Tajikistan) and south of the Pamir range (Himalayan ibex) (Figure 2A). Undoubtedly, Siberian ibex are the undisputed master of terrifying heights and known for their rock-climbing skills, as they are often sighted on the pinnacles of mountain tops [70]. However, they are not so well adapted to cross large rivers and, notably, Indus and the Amu Darya are the two major rivers in the Trans-Himalayan region of South and Central Asia that likely impacted, along with the other factors, in historic divergence and contemporary gene flow in Siberian ibex. The Indus River originates in Western Tibet and flows northwest through the Ladakh and Gilgit-Baltistan regions of Kashmir, bends sharply to the left after the Nanga Parbat massif, and flows south-by-southwest through Pakistan [71]. Meanwhile, the Amu Darya river rises in the Pamir Mountains, north of the Hindu Kush, and flows from there northwestwards into the southern remnants of the Aral Sea [72]. In its upper stream, the river forms part of Afghanistan’s northern border with Tajikistan, Uzbekistan, and Turkmenistan. Most high mountains in the Pamir knot run from an east to west direction, transversely bisected by these river networks (Figure 3). Hence, we presume that the simultaneous and steady flow of these rivers along the side of high mountains possibly hindered the continuous movement of Siberian ibex in the Pamir landscape along with the other miscellaneous factors.

In addition, we also testified whether the river network disrupted the gene flow in Himalayan ibex and, interestingly, we obtained distinct haplotypes on the northern and southern side of the Indus River in the ITHR (Figure S2). This observation, though, requires intensive sampling but provides probable patterns showing the river system restricting the gene flow in Himalayan ibex at least to some extent.

## 5. Conclusions

The present study provides pragmatic genomic evidence of allopatric speciation and deep divergence of Himalayan ibex from the main range of Siberian ibex following PSPC. Together with the large climatic episodes and geological events mingled with the likely effect of river systems contributed to the wide separation of ibex populations in and around the Pamir Mountains. In this context, the IUCN recent assessment highlights a declining population trend of *C. sibirica* at large [16], while ibex in India already had restricted distribution in the Trans-Himalayas and its wild populations are highly vulnerable due to habitat loss, illegal poaching, human disturbance, and dietary competition with livestock [16]. Findings of the present study will allow us to identify the hitherto unreported consequences of past geo-climatic events in Asian highlands, and open up opportunities to test the consistency of the PSPC across various ungulates like Tibetan gazelle (*Procapra picticaudata*), Tibetan antelope (*Pantholops hodgsoni*), blue sheep (*Pseudois nayaur*), Ladakh urial (*Ovis vignei vignei*), Tibetan argali (*Ovis ammon hodgsoni*), Tibetan wild ass (*Equus kiang*), and wild yak (*Bos grunniens*) distributed in this region [73,74]. We propose that Himalayan ibex is an evolutionary distinct phylogenetic species of Siberian ibex, and it shall be worthy to upgrade its taxonomic status as *Capra himalayensis* in order to attract concentrated efforts for conservation planning at regional as well as global levels. In conclusion, we are eager to expand our study by including additional genomes from Siberian ibex populations across various ranges, such as those from Pakistan, Russia, Mongolia, Altai mountains, and beyond. By doing so, we aim to gain a more comprehensive understanding of the detailed phylogeographic signature and population structure, encompassing range-wide samples.

We anticipate that the results of our extended research will draw the attention of biologists and conservationists studying ibex in a diverse range of countries. We hope that this will stimulate the initiation of multi-lateral, multi-national, and cross-cutting dialogues focused on the conservation and management of global ibex populations. By fostering collaborative efforts and data-sharing initiatives, we envision a synergistic approach to address conservation challenges and implement effective management strategies for the preservation of this magnificent species.

## Data Availability

The data underlying this article are available in the NCBI-SRA database at https://www.ncbi.nlm.nih.gov/ (accessed on 20 August 2021), which can be accessed with Bio-Project Accession: PRJNA760289. All the source of data analysis software and genomic data is provided in Supplementary Table S7.

## Supplementary Materials

The following are available online at https://www.mdpi.com/article/10.3390/biology12081097/s1, Figure S1. Bayesian-based phylogenetic relationship of four species of ibex under the genus *Capra* performed using BEAUti v 1.6.1 and BEAST v.2.10.4. Figure S2. Phylogeographic distribution of different haplotypes among four species of ibex based on median joining network. The Himalayan ibex formed a paraphyletic clade within Siberian ibex supporting genetic divergence/allopatric speciation in Himalayan ibex. Further, haplotypes observed in Himalayan ibex are also plotted over the map overlaying river system network that represented three haplotypes (H29, H30 and H32) found in the north side of Indus river and five haplotypes (H25, H26, H27, H28 and H31) found in the south side of the Indus river. Figure S3. Principal component analysis (PCA) derived by the genomes of Siberian ibex and Himalayan ibex. Figure S4. Tree-Mix models by allowing 0-6 migrations with residuals suggested complex admixture among populations from *C.sibirica, C.ibex, C.pyrenaica* and *C.Nubiana*. Figure S5 Digital elevation model of the landscape. (A) present; (B) PaleoDEM (around 3MYA). Figure S6 Frequency distribution of elevation within the area of interest (Red = Selected bins of cutoff).(A). present; (B). past around 3MYA. Table S1. GenBank/NCBI accessions of the four species of ibex under the genus *Capra* used in the present study. Table S2. Haplotype information and GeneBank/NCBI accessions of cytochrome b gene of the four ibex species under the genus *Capra* used in the present study. Table S3. Score value of D-statistics keeping Himalayan ibex at H3 shows greater affinity between H1 & H2. Table S4. Score value of D-statistics keeping Himalayan ibex at H3 shows greater affinity between H1 & H3. Table S5. Comparative assessment of elevation profile within the specific area of interest (India-Tajikistan) between present and paleo DEMs (3MYA). Table S6. Key Resources Table. Table S7. Descriptive statistics of elevation profiles within the area of interest between present and paleo DEMs (3MYA).
