# Supplementary material for: Mid-Pleistocene Transitions Forced Himalayan ibex to Evolve Independently after Split into an Allopatric Refugium
Source: Biology (Basel). 2023 Aug 7;12(8):1097. doi: 10.3390/biology12081097 (PMC10451794; doi:10.3390/biology12081097)
Supplement: Supplementary file 1 [file biology-12-01097-s001.zip › biology-2486275-supplementary.pdf]

biology

Supplementary Information for Mid-Pleistocene Transitions Forced Himalayan ibex to Evolve Independently after Split into an Allopatric Refugium

Gul Jabin [1,2], Bheem Dutt Joshi [1], Ming-Shan Wang [3], Tanoy Mukherjee [1], Stanzin Dolker [1,2], Sheng Wang [4], Kailash Chandra [1], Venkatraman Chinnadurai [5], Lalit Kumar Sharma [1] and Mukesh Thakur [1,*]

[1] Zoological Survey of India, Kolkata 700053, India
[2] Department of Zoology, University of Calcutta, Kolkata 700019, India
[3] Howard Hughes Medical Institute, University of California Santa Cruz, Santa Cruz, CA 95064, USA
[4] Kunming Institute of Zoology, Kunming 650223, China
[5] Zoological Survey of India, Marine Biology Regional Centre (MBRC), Chennai 600028, India
* Correspondence: author: thamukesh@gmail.com

Figures and Tables

Figure S1. Bayesian-based phylogenetic relationship of four species of ibex under the genus *Capra* performed using BEAUti v 1.6.1 and BEAST v.2.10.4 (Suchard et al., 2018)........ 3
Figure S2 Phylogeographic distribution of different haplotypes among four species of ibex based on median joining network. The Himalayan ibex formed a paraphyletic clade within Siberian ibex supporting genetic divergence / allopatric speciation in Himalayan ibex. Further, haplotypes observed in Himalayan ibex are also plotted over the map overlaying river system network that represented three haplotypes (H29, H30 and H32) found in the north side of Indus river and five haplotypes (H25, H26, H27, H28 and H31) found in the south side of the Indus river... 4
Figure S3 Principal component analysis (PCA) derived by the genomes of Siberian ibex and Himalayan ibex. 5
Figure S4 Tree-Mix models by allowing 0-6 migrations with residuals suggested complex admixture among populations from *C.sibirica, C.ibex, C.pyrenaica* and *C.Nubiana* 6
Figure S5 Digital elevation model of the landscape. (A) present; (B) PaleoDEM (around 3MYA) 7
Figure S6 Frequency distribution of elevation within the area of interest (Red=Selected bins of cutoff).(A). present; (B). past around 3MYA 8

Table S1. GenBank/NCBI accessions of the four species of ibex under the genus *Capra* used in the present study. 8
Table S2 Haplotype information and GeneBank/NCBI accessions of cytochrome b gene of the four ibex species under the genus *Capra* used in the present study........ 9
Table S3. Score value of D-statistics keeping Himalayan ibex at H3 shows greater affinity between H1 & H2 12
Table S4. Score value of D-statistics keeping Himalayan ibex at H3 shows greater affinity between H1 & H3 21
Table S5. Comparative assessment of elevation profile within the specific area of interest (India- Tajikistan) between present and paleo DEMs (3MYA) 23
Table S6 Key Resources Table 24
Table S7 Descriptive statistics of elevation profiles within the area of interest between present and paleo DEMs (3MYA)........ 26

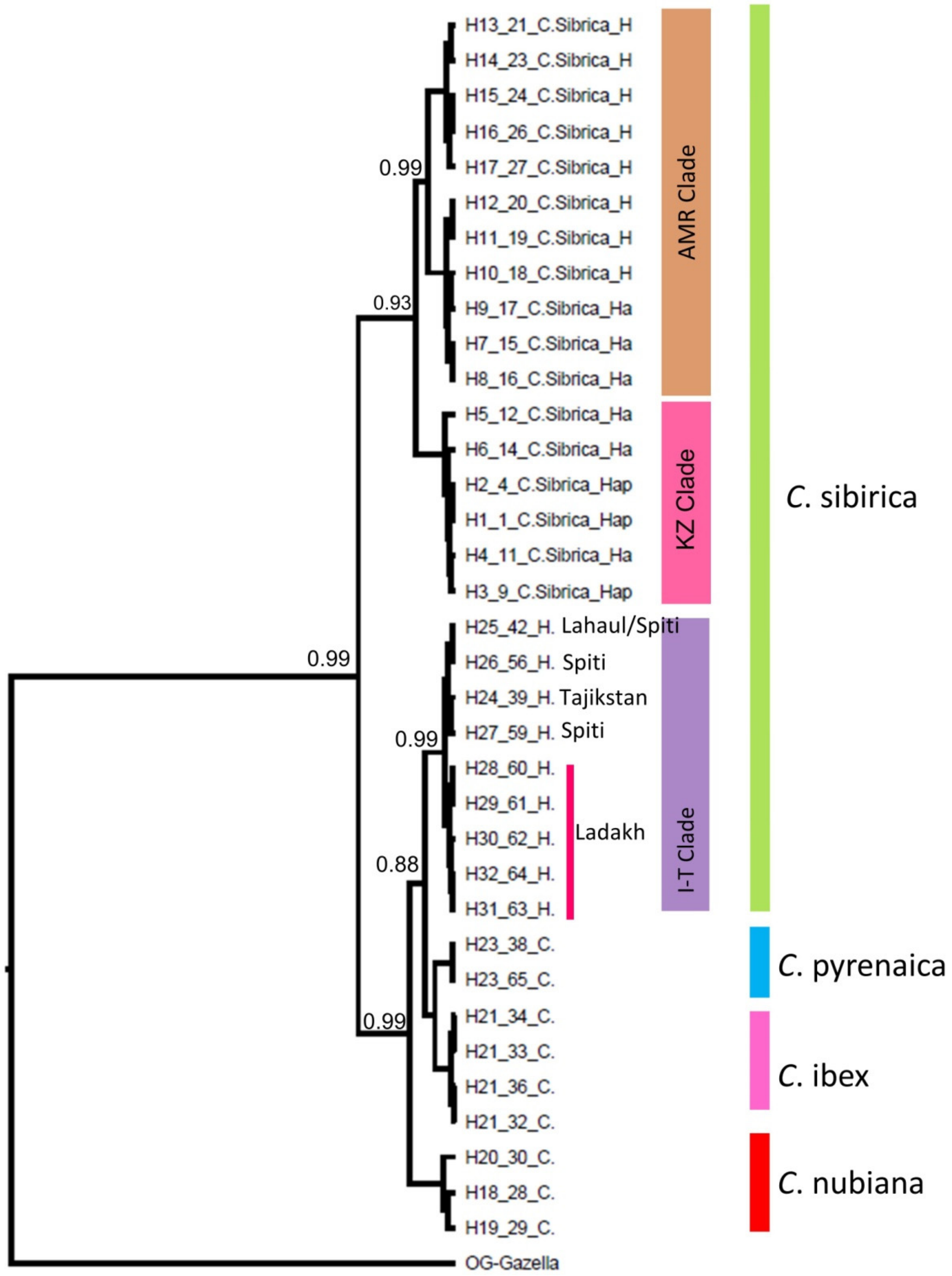

Figure S1. Bayesian-based phylogenetic relationship of four species of ibex under the genus *Capra* performed using BEAUti v 1.6.1 and BEAST v.2.10.4 (Suchard et al., 2018).

Following our previous study (Joshi et al., 2020), samples of Himalayan ibex placed as a paraphyletic clade (I-T clade) within *C. sibirica* supporting genetic divergence/allopatric speciation in Himalayan ibex.

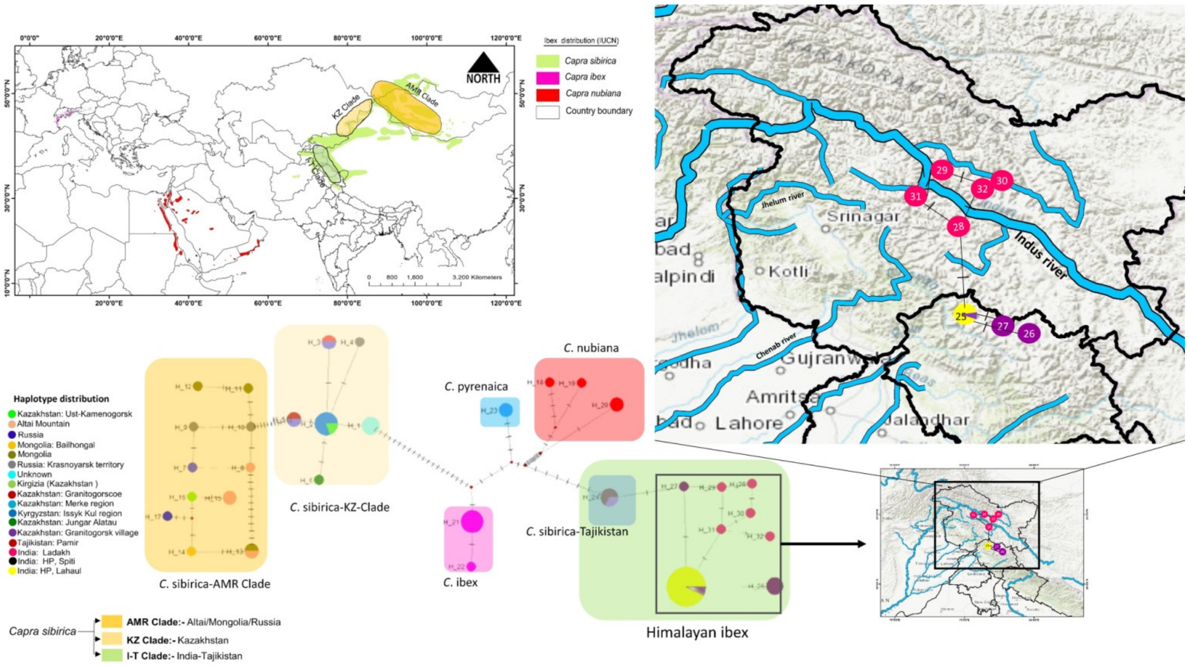

Figure S2. Phylogeographic distribution of different haplotypes among four species of ibex based on median joining network. The Himalayan ibex formed a paraphyletic clade within Siberian ibex supporting genetic divergence/allopatric speciation in Himalayan ibex. Further, haplotypes observed in Himalayan ibex are also plotted over the map overlaying river system network that represented three haplotypes (H29, H30, and H32) found on the north side of Indus River and five haplotypes (H25, H26, H27, H28, and H31) found on the south side of the Indus River. .

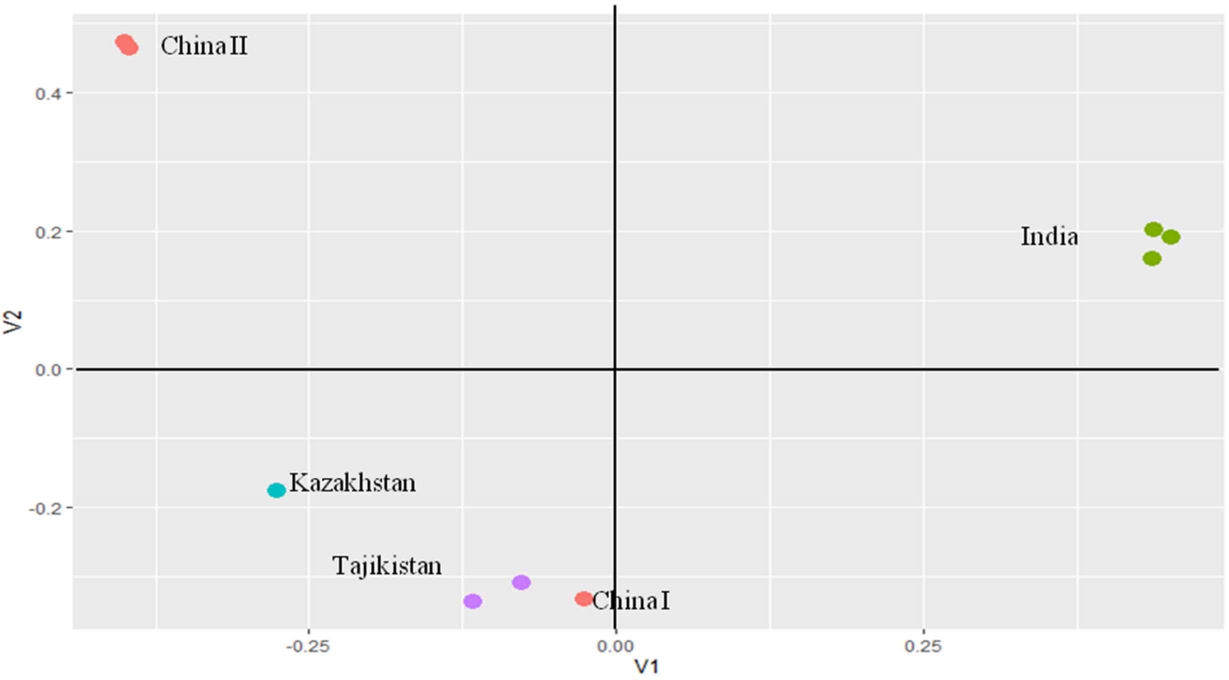

Figure S3. Principal component analysis (PCA) derived by the genomes of Siberian ibex and Himalayan ibex.

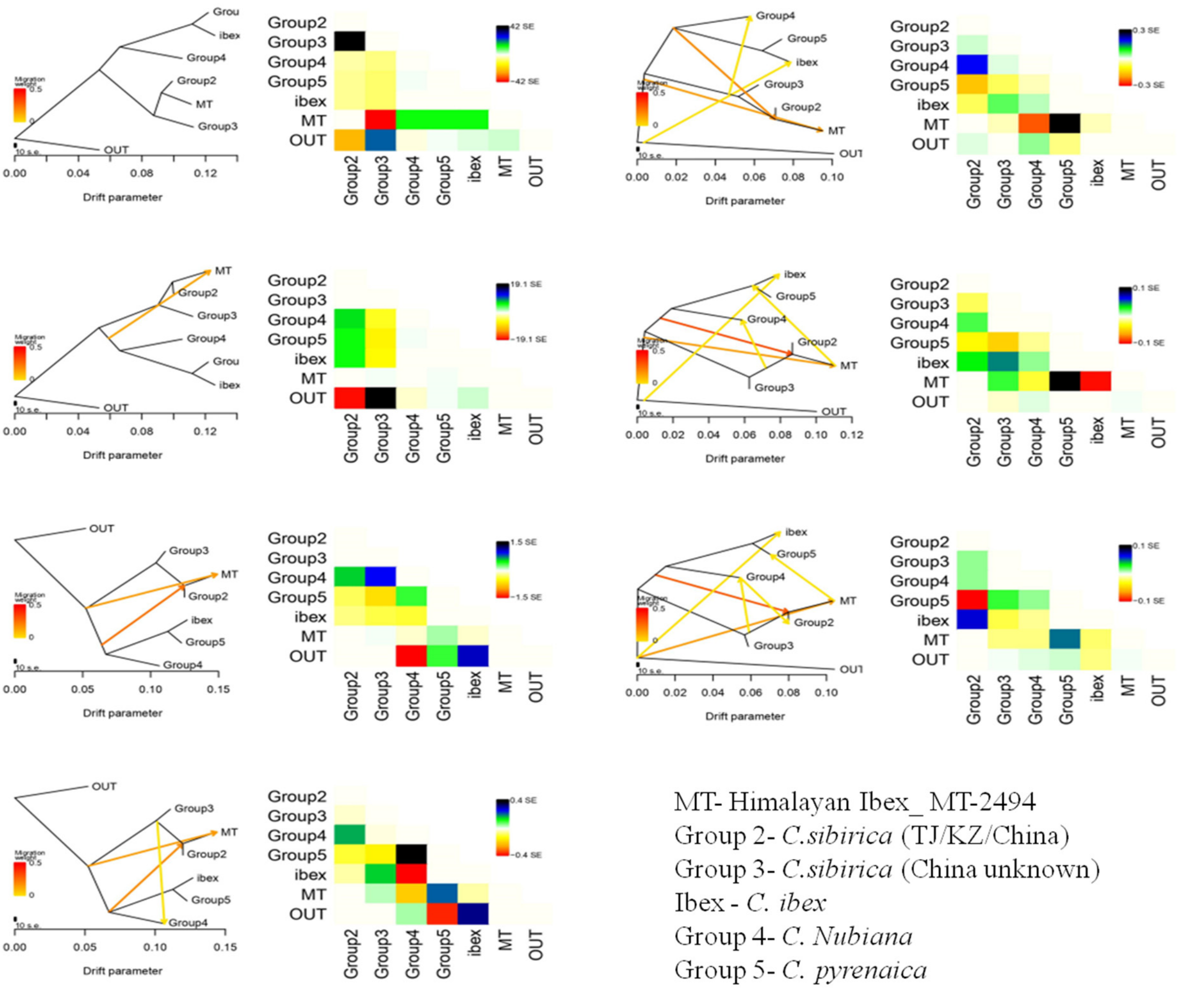

Figure S4. Tree-Mix models allowing 0-6 migrations with residuals suggested complex admixture among populations from *C. sibirica*, *C. ibex*, *C. pyrenaica*, and *C. nubiana*.

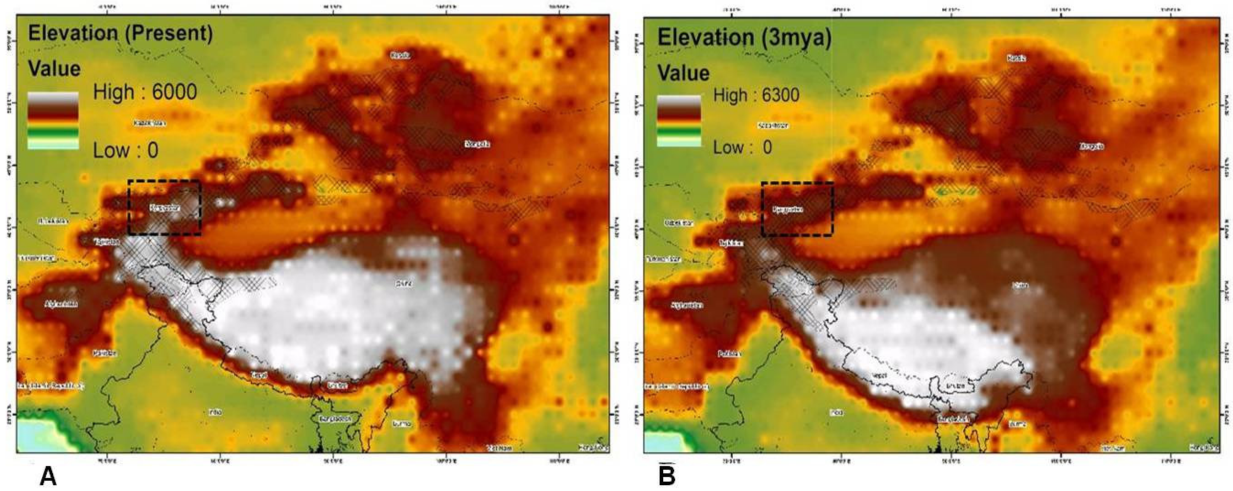

Figure S5. Digital elevation model of the landscape. (A) Present; (B) PaleoDEM (around 3 MYA).

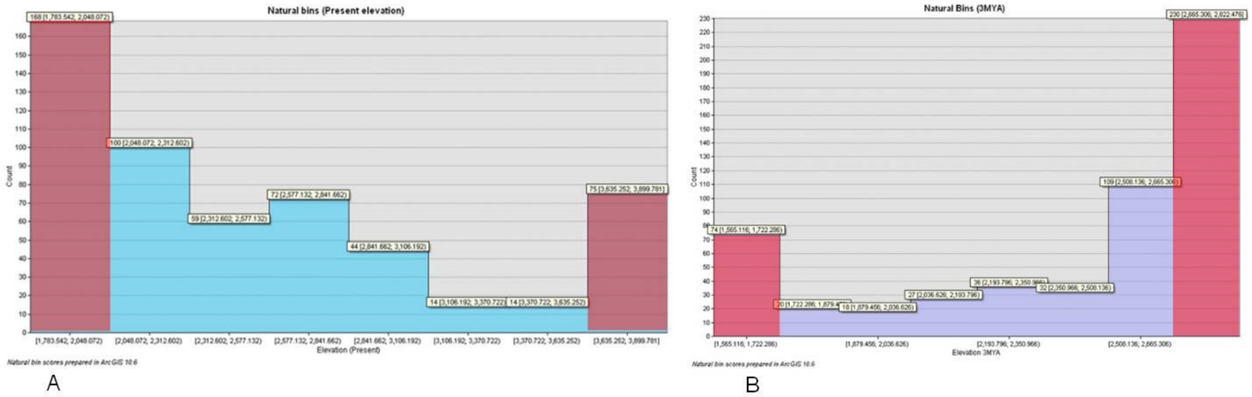

Figure S6. Frequency distribution of elevation within the area of interest (red = selected bins of cut-off). (A) Present; (B) past around 3 MYA.

Table S1. GenBank/NCBI accessions of the four species of ibex under the genus *Capra* used in the present study.

S.No	Population	ACCESSION ID /ZSI ID	SPECIES	REGION/Population	DATA SIZE
1	Pop.1	SRR8437784	*Capra sibirica (11)*	Tajikistan	R1=20.7 GB, R2=23.4 GB
2	Pop.1	ERR4133453	*Capra sibirica*	Tajikistan	R1=9.4 GB, R2=11.2GB
3	Pop.2	SRR5803200	*Capra sibirica*	China ecotype	R1=7.1GB, R2=5.1 GB
4	Pop.2	SRR5803204	*Capra sibirica*	China ecotype	R1=8.2 GB, R2=9.3 GB
5	Pop.3	SRR8437786	*Capra sibirica*	Kazakhstan	R1= 17.7 GB, R2=16.8 GB
6	Pop.4	MT-2414 PRJNA760289	*Capra sibirica*	Lahaul, Himachal, Pradesh, India	R1=6.8GB, R2=6.9GB
7	Pop.4	MT-2415 PRJNA760289	*Capra sibirica*	Ladakh, India	R1=7.0GB, R2= 7.1GB
8	Pop.4	MT-2494 PRJNA760289	*Capra sibirica*	Ladakh, India	R1= 39.1, GB,R2=38.1GB
9	Pop.5	SRR5803192	*Capra sibirica*	China ecotype	R1=16.6 GB, R2=18.9 GB
10	Pop.5	SRR5803193	*Capra sibirica*	China ecotype	R1=7.1GB, R2=5.1 GB
11	Pop.6	SRR8437792	*Capra nubiana (03)*	Egypt	R1=20.9 GB, R2=23 GB
12	Pop.6	ERR4133452	*Capra nubiana*	Israel	R1=9.2 GB, R2=10.9 GB
13	Pop.7	SRR8437789	*Capra nubiana*	Central Saudi Arabia	R1=17.5 GB, R2=20 GB
14	Pop.8	SRR8437787	*Capra pyrenaica (05)*	Sierra de Gredos, Spain	R1=15.9 GB, R2=17.3 GB
15	Pop.8	SRR8437788	*Capra pyrenaica*	Sierra de Gredos, Spain	R1=15.3 MB, R2=16.8 GB
16	Pop.8	SRR8437790	*Capra pyrenaica*	Spain	R1=24.8 GB, R2=28 GB
17	Pop.9	ERR4133262	*Capra pyrenaica*	Museum, France	R1=19 GB, R2=21.3 GB
18	Pop.10	SRR8437785	*Capra pyrenaica*	Maestrazgo, Spain	R1=16.1 GB, R2=17.8 GB
19	Pop.11	ERR4133400	*Capra ibex (21)*	Italy	R1=4.8 GB, R2=5.4 GB
20	Pop.12	SRR8437793	*Capra ibex*	Grand Paradiso	R1=15.9 GB, R2=17.7 GB
21	Pop.12	SRR8437808	*Capra ibex*	Weisshorn	R1=18.9 GB, R2=20 GB
22	Pop.13	SRR5803190	*Capra ibex*	China ecotype	R1=13.1 GB, R2=13.4 GB
23	Pop.14	SRR8437774	*Capra ibex*	Alpi maritime	R1=15.7 GB, R2=18.2 GB
24	Pop.14	SRR8437780	*Capra ibex*	Alpi maritime	R1=15.6 GB, R2=18.1 GB
25	Pop.14	SRR8437805	*Capra ibex*	Weisshorn	R1=15.8 GB, R2=17.8 GB
26	Pop.15	SRR8437801	*Capra ibex*	Albris	R1=17.3 GB, R2=18.2 GB
27	Pop.15	SRR8437810	*Capra ibex*	Albris	R1=17.2 GB, R2=18.9 GB
28	Pop.15	SRR8437811	*Capra ibex*	Brienzer Rothorn	R1=17.9 GB, R2=20 GB
29	Pop.16	SRR8437775	*Capra ibex*	Brienzer Rothorn	R1=18.3 GB, R2=20.3 GB
30	Pop.16	SRR8437777	*Capra ibex*	Bire Oeschinen	R1=16.9 GB, R2=18.7 GB
31	Pop.16	SRR8437800	*Capra ibex*	Rheinwaldhorn	R1=16.5 GB, R2=17.4 GB
32	Pop.17	SRR8437796	*Capra ibex*	Grand Paradiso	R1=17.7 GB, R2=19.1 GB
33	Pop.17	SRR8437806	*Capra ibex*	Oberbauerstock	R1=17.3 GB, R2=19.3 GB

34	Pop.17	ERR4133450	*Capra ibex*	Italy	R1=10.3 GB, R2=12.1 GB
35	Pop.17	SRR8437782	*Capra ibex*	Bire Oeschinen	R1=15.8 GB, R2=17.4 GB
36	Pop.17	SRR8437791	*Capra ibex*	Pleurer	R1=15.4 GB, R2=17.3 GB
37	Pop.17	SRR8437803	*Capra ibex*	Pleurer	R1=17.3 GB, R2=19.5 GB
38	Pop.17	SRR8437809	*Capra ibex*	Oberbauerstock	R1=16.3 GB, R2=17.9 GB
39	Pop.17	SRR8437812	*Capra ibex*	Oberbauerstock	R1=11.2 GB, R2=11.4 GB
40	-	ERR2214953	*C. aegagrus*		
41	-	SRR8437778	*Capra falconeri*		

Table S2. Haplotype information and GenBank/NCBI accessions of cytochrome b gene of the four ibex species under the genus *Capra* used in the present study.

S. no.	Hap ID	GenBank ID	Species	Location
1	H1	FJ207529.1	*C. sibirica*	Unknown
2	H1	AF034734.1	*C. sibirica*	Unknown
3	H1	NC 020626.1	*C. sibirica*	Unknown
4	H2	KY366505.1	*C. sibirica*	Kyrgyzstan: Issyk-Kul region
5	H2	KY366504.1	*C. sibirica*	Kyrgyzstan: Issyk-Kul region
6	H2	DQ246773.1	*C. sibirica*	Altai Mountain
7	H2	DQ246770.1	*C. sibirica*	Altai Mountain
8	H2	KT633871.1	*C. sibirica*	Kyrgyzstan: Issyk-Kul region
9	H3	KY366503.1	*C. sibirica*	Kazakhstan: Granitogorsk village
10	H3	KT246294.1	*C. sibirica*	Kazakhstan: Granitogorscoe
11	H4	CDQ246772.	*C. sibirica*	Altai Mountain
12	H5	KT633870.	*C. sibirica*	Kazakhstan: Granitogorscoe
13	H5	KT283245.	*C. sibirica*	Kazakhstan: Merke region
14	H6	KX096852.	*C. sibirica*	Kazakhstan: Jungar Alatau
15	H7	KY491664.1	*C. sibirica*	Russia*
16	H8	DQ246775.1	*C. sibirica*	Altai Mountain
17	H9	KX096853.	*C. sibirica*	Russia: Krasnoyarsk region
18	H10	KT283242.	*C. sibirica*	Russia: Krasnoyarsk territory, Ermakovski
19	H11	DQ514551.1	*C. sibirica*	Mongolia
20	H12	KF990328.	*C. sibirica*	Mongolia
21	H13	DQ514550.1	*C. sibirica*	Mongolia
22	H13	DQ246774.1	*C. sibirica*	Altai Mountain
23	H14	KT260194.	*C. sibirica*	Mongolia: district Bailhongal
24	H15	DQ246779.1	*C. sibirica*	Altai Mountain
25	H15	DQ246771.1	*C. sibirica*	Altai Mountain
26	H16	KX096851.	*C. sibirica*	Kazakhstan: Ust-Kamenogorsk
27	H17	KU165679.	*C. sibirica*	Russia*
28	H24	KY996521.1	*C. sibirica*	Tajikistan: Pamir
29	H24	KX910792.1	*C. sibirica*	Tajikistan: Pamir
30	H24	DQ246799.1	*C. sibirica*	Russia
31	H25	MN720106	*C. sibirica* (Himalayan ibex)	Lahaul, Miyar
32	H25	MN720106	*C. sibirica* (Himalayan ibex)	Lahaul, Kurched
33	H25	MN720106	*C. sibirica* (Himalayan ibex)	Spiti, Kibber, WL
34	H25	MN720106	*C. sibirica* (Himalayan ibex)	Lahaul, Udaipur
35	H25	MN720106	*C. sibirica* (Himalayan ibex)	Lahaul, Jhalma
36	H25	MN720106	*C. sibirica* (Himalayan ibex)	Lahaul, Jhalma
37	H25	MN720106	*C. sibirica* (Himalayan ibex)	Lahaul, Jhalma
38	H25	MN720106	*C. sibirica* (Himalayan ibex)	Lahaul, Udaipur
39	H25	MN720106	*C. sibirica* (Himalayan ibex)	Lahaul, Miyar
40	H25	MN720106	*C. sibirica* (Himalayan ibex)	Lahaul, Udaipur
41	H25	MN720106	*C. sibirica* (Himalayan ibex)	Lahaul, Jhalma
42	H25	MN720106	*C. sibirica* (Himalayan ibex)	Lahaul, Udaipur
43	H25	MN720106	*C. sibirica* (Himalayan ibex)	piti, KibberWLS, Khanamau
44	H25	MN720106	*C. sibirica* (Himalayan ibex)	Lahaul, Miyar

45	H26	MN720107	*C. sibirica* (Himalayan ibex)	Spiti, KibberWL
46	H26	MN720107	*C. sibirica* (Himalayan ibex)	Spiti, Kibber
47	H26	MN720107	*C. sibirica* (Himalayan ibex)	Spiti, Kibber, Ladarcha
48	H27	UK315797	*C. sibirica* (Himalayan ibex)	Spiti, Kibber
49	H28	UK315798	*C. sibirica* (Himalayan ibex)	Ladakh, India
50	H29	UK315799	*C. sibirica* (Himalayan ibex)	Ladakh, India
51	H30	UK315800	*C. sibirica* (Himalayan ibex)	Ladakh, India
52	H31	UK315801	*C. sibirica* (Himalayan ibex)	Ladakh, India
53	H32	UK315802	*C. sibirica* (Himalayan ibex)	Ladakh, India
54	H18	AF217256.1	*C. nubiana*	Unknown
55	H19	DQ514552.1	*C. nubiana*	Unknown
56	H20	NC_020624.1	*C. nubiana*	Unknown
57	H20	FJ207527.1	*C. nubiana*	Unknown
58	H21	FJ207526.1	*C. ibex*	Unknown
59	H21	SRR8437775	*C. ibex*	Unknown
60	H21	SRR8437774.	*C. ibex*	Unknown
61	H21	MF139817.1	*C. ibex*	Unknown
62	H21	MF139798.1	*C. ibex*	Unknown
63	H22	MF139808.1	*C. ibex*	Unknown
64	H23	SRX5245273	*C. pyrenaica*	Unknown
65	H24	FJ207528.1	*C. pyrenaica*	Unknown
66	-----	KM523443.1	*Gazella gazella*	Outgroup

Table S3. Score value of D-statistics keeping Himalayan ibex at H3 shows greater affinity between H1 and H2.

H1	H2	H3	nABBA	nBABA	Dstat	SE	Z
G2.ERR4133453_CS-TJ	G3.SRR5803192_CS-China II	MT_2494	818375	1345855	-0.243726	0.0032232	-75.61657
G2.ERR4133453_CS-TJ	G3.SRR5803192_CS-China II	MT-2414	791836	1327683	-0.252815	0.0032707	-77.29626
G2.ERR4133453_CS-TJ	G3.SRR5803192_CS-China II	MT-2415	798824	1364194	-0.26138	0.0032962	-79.29662
G2.ERR4133453_CS-TJ	G3.SRR5803193_CS-China II	MT_2494	828530	1397616	-0.255637	0.0031601	-80.89443
G2.ERR4133453_CS-TJ	G3.SRR5803193_CS-China II	MT-2414	796618	1374300	-0.2661	0.0030499	-87.2492
G2.ERR4133453_CS-TJ	G3.SRR5803193_CS-China II	MT-2415	803323	1412731	-0.274997	0.0031954	-86.06075
G2.ERR4133453_CS-TJ	G4.SRR8437789_C.Nubiana	MT_2494	656090	3630030	-0.693854	0.0025011	-277.415
G2.ERR4133453_CS-TJ	G4.SRR8437789_C.Nubiana	MT-2414	614275	3568172	-0.706261	0.0022626	-312.1411
G2.ERR4133453_CS-TJ	G4.SRR8437789_C.Nubiana	MT-2415	602323	3641253	-0.716125	0.0022743	-314.8774
G2.ERR4133453_CS-TJ	G4.SRR8437792_C.Nubiana	MT_2494	656069	3653492	-0.695529	0.0025327	-274.6214
G2.ERR4133453_CS-TJ	G4.SRR8437792_C.Nubiana	MT-2414	616944	3588988	-0.706632	0.0024274	-291.1101
G2.ERR4133453_CS-TJ	G4.SRR8437792_C.Nubiana	MT-2415	603974	3661986	-0.71684	0.0023319	-307.4082
G2.ERR4133453_CS-TJ	G5.SRR8437785_C.Pyrenaica	MT_2494	654925	3619835	-0.693585	0.0025958	-267.2
G2.ERR4133453_CS-TJ	G5.SRR8437785_C.Pyrenaica	MT-2414	615485	3559583	-0.705162	0.0024073	-292.9285
G2.ERR4133453_CS-TJ	G5.SRR8437785_C.Pyrenaica	MT-2415	602893	3633151	-0.715351	0.0024443	-292.6586
G2.ERR4133453_CS-TJ	G5.SRR8437787_C.Pyrenaica	MT_2494	652339	3637126	-0.695841	0.0024961	-278.7678
G2.ERR4133453_CS-TJ	G5.SRR8437787_C.Pyrenaica	MT-2414	614521	3576391	-0.706736	0.0023671	-298.5619
G2.ERR4133453_CS-TJ	G5.SRR8437787_C.Pyrenaica	MT-2415	602141	3649688	-0.716761	0.0024388	-293.8977
G2.ERR4133453_CS-TJ	G5.SRR8437788_C.Pyrenaica	MT_2494	652597	3637901	-0.695794	0.0025909	-268.5555
G2.ERR4133453_CS-TJ	G5.SRR8437788_C.Pyrenaica	MT-2414	615082	3577013	-0.706552	0.0024071	-293.5301
G2.ERR4133453_CS-TJ	G5.SRR8437788_C.Pyrenaica	MT-2415	602804	3648591	-0.716421	0.0024408	-293.5193
G2.ERR4133453_CS-TJ	G5.SRR8437790_C.Pyrenaica	MT_2494	658097	3621743	-0.692467	0.0026899	-257.4305
G2.ERR4133453_CS-TJ	G5.SRR8437790_C.Pyrenaica	MT-2414	615813	3566487	-0.705515	0.0023531	-299.8176
G2.ERR4133453_CS-TJ	G5.SRR8437790_C.Pyrenaica	MT-2415	603770	3638456	-0.715352	0.0024758	-288.9332
G2.SRR5803200_CS-China	G4.SRR8437789_C.Nubiana	MT_2494	625379	3660557	-0.708172	0.0023104	-306.5152
G2.SRR5803200_CS-China	G4.SRR8437789_C.Nubiana	MT-2414	590125	3600801	-0.71838	0.0021297	-337.3172
G2.SRR5803200_CS-China	G4.SRR8437789_C.Nubiana	MT-2415	578533	3677463	-0.728133	0.0021442	-339.587

G2.SRR5803200_CS-China	G4.SRR8437792_C.Nubiana	MT_2494	624751	3682760	-0.709925	0.0023429	-303.0053
G2.SRR5803200_CS-China	G4.SRR8437792_C.Nubiana	MT-2414	590537	3619511	-0.719463	0.0022124	-325.1917
G2.SRR5803200_CS-China	G4.SRR8437792_C.Nubiana	MT-2415	578927	3697088	-0.729221	0.0021926	-332.5766
G2.SRR5803200_CS-China	G5.SRR8437785_C.Pyrenaica	MT_2494	626399	3649940	-0.70704	0.0024203	-292.1262
G2.SRR5803200_CS-China	G5.SRR8437785_C.Pyrenaica	MT-2414	591403	3590554	-0.717165	0.0022515	-318.5337
G2.SRR5803200_CS-China	G5.SRR8437785_C.Pyrenaica	MT-2415	580481	3669403	-0.726825	0.0023232	-312.8546
G2.SRR5803200_CS-China	G5.SRR8437787_C.Pyrenaica	MT_2494	623483	3668443	-0.709462	0.0023391	-303.309
G2.SRR5803200_CS-China	G5.SRR8437787_C.Pyrenaica	MT-2414	590478	3609227	-0.7188	0.0021961	-327.3032
G2.SRR5803200_CS-China	G5.SRR8437787_C.Pyrenaica	MT-2415	579901	3687130	-0.728195	0.0023017	-316.3765
G2.SRR5803200_CS-China	G5.SRR8437788_C.Pyrenaica	MT_2494	624341	3668757	-0.709142	0.0024311	-291.6951
G2.SRR5803200_CS-China	G5.SRR8437788_C.Pyrenaica	MT-2414	591559	3609578	-0.718382	0.0022564	-318.3739
G2.SRR5803200_CS-China	G5.SRR8437788_C.Pyrenaica	MT-2415	581291	3686769	-0.727609	0.0023546	-309.0121
G2.SRR5803200_CS-China	G5.SRR8437790_C.Pyrenaica	MT_2494	629348	3652828	-0.706062	0.0025263	-279.4888
G2.SRR5803200_CS-China	G5.SRR8437790_C.Pyrenaica	MT-2414	591757	3598962	-0.717587	0.0022199	-323.2467
G2.SRR5803200_CS-China	G5.SRR8437790_C.Pyrenaica	MT-2415	581495	3675582	-0.72681	0.0023383	-310.828
G2.SRR5803204_CS-China	G4.SRR8437789_C.Nubiana	MT_2494	635905	3669212	-0.704582	0.002354	-299.3081
G2.SRR5803204_CS-China	G4.SRR8437789_C.Nubiana	MT-2414	598231	3609561	-0.715656	0.0022179	-322.6686
G2.SRR5803204_CS-China	G4.SRR8437789_C.Nubiana	MT-2415	584756	3689205	-0.726363	0.0022157	-327.8325
G2.SRR5803204_CS-China	G4.SRR8437792_C.Nubiana	MT_2494	635329	3691485	-0.706329	0.0023615	-299.0987
G2.SRR5803204_CS-China	G4.SRR8437792_C.Nubiana	MT-2414	599730	3628956	-0.716352	0.0022923	-312.4977
G2.SRR5803204_CS-China	G4.SRR8437792_C.Nubiana	MT-2415	585626	3708988	-0.727274	0.0022747	-319.717
G2.SRR5803204_CS-China	G5.SRR8437785_C.Pyrenaica	MT_2494	635565	3658461	-0.703977	0.0023712	-296.8876
G2.SRR5803204_CS-China	G5.SRR8437785_C.Pyrenaica	MT-2414	599513	3601400	-0.71458	0.0023367	-305.8036
G2.SRR5803204_CS-China	G5.SRR8437785_C.Pyrenaica	MT-2415	586873	3681102	-0.724988	0.0023792	-304.7144
G2.SRR5803204_CS-China	G5.SRR8437787_C.Pyrenaica	MT_2494	634457	3676889	-0.70568	0.0022761	-310.046
G2.SRR5803204_CS-China	G5.SRR8437787_C.Pyrenaica	MT-2414	598899	3618483	-0.715985	0.0022451	-318.908
G2.SRR5803204_CS-China	G5.SRR8437787_C.Pyrenaica	MT-2415	587428	3698494	-0.72588	0.0023876	-304.0182
G2.SRR5803204_CS-China	G5.SRR8437788_C.Pyrenaica	MT_2494	635438	3677912	-0.705362	0.002397	-294.2663
G2.SRR5803204_CS-China	G5.SRR8437788_C.Pyrenaica	MT-2414	599952	3619178	-0.715604	0.002315	-309.1161

G2.SRR5803204_CS-China	G5.SRR8437788_C.Pyrenaica	MT-2415	588764	3697568	-0.725283	0.0024555	-295.3674
G2.SRR5803204_CS-China	G5.SRR8437790_C.Pyrenaica	MT_2494	640002	3661769	-0.702447	0.0025376	-276.8198
G2.SRR5803204_CS-China	G5.SRR8437790_C.Pyrenaica	MT-2414	600048	3608982	-0.714876	0.0022746	-314.2853
G2.SRR5803204_CS-China	G5.SRR8437790_C.Pyrenaica	MT-2415	588946	3687638	-0.724572	0.002496	-290.2901
G2.SRR8437784_CS-TJ	G4.SRR8437789_C.Nubiana	MT_2494	674644	3605456	-0.684753	0.0026889	-254.6551
G2.SRR8437784_CS-TJ	G4.SRR8437789_C.Nubiana	MT-2414	632997	3522433	-0.69534	0.0023947	-290.367
G2.SRR8437784_CS-TJ	G4.SRR8437789_C.Nubiana	MT-2415	622918	3589206	-0.704226	0.0024437	-288.1806
G2.SRR8437784_CS-TJ	G4.SRR8437792_C.Nubiana	MT_2494	674053	3628209	-0.686652	0.0026954	-254.7451
G2.SRR8437784_CS-TJ	G4.SRR8437792_C.Nubiana	MT-2414	634352	3542409	-0.696247	0.0024935	-279.2267
G2.SRR8437784_CS-TJ	G4.SRR8437792_C.Nubiana	MT-2415	622982	3608466	-0.705547	0.0024608	-286.7118
G2.SRR8437784_CS-TJ	G5.SRR8437785_C.Pyrenaica	MT_2494	672781	3593066	-0.684573	0.0027308	-250.6887
G2.SRR8437784_CS-TJ	G5.SRR8437785_C.Pyrenaica	MT-2414	632069	3511288	-0.6949	0.0024979	-278.1948
G2.SRR8437784_CS-TJ	G5.SRR8437785_C.Pyrenaica	MT-2415	622660	3578778	-0.703597	0.0025731	-273.4484
G2.SRR8437784_CS-TJ	G5.SRR8437787_C.Pyrenaica	MT_2494	670007	3610364	-0.68694	0.0026099	-263.2084
G2.SRR8437784_CS-TJ	G5.SRR8437787_C.Pyrenaica	MT-2414	631226	3528338	-0.696494	0.0024341	-286.1422
G2.SRR8437784_CS-TJ	G5.SRR8437787_C.Pyrenaica	MT-2415	622014	3596124	-0.705077	0.0025446	-277.0832
G2.SRR8437784_CS-TJ	G5.SRR8437788_C.Pyrenaica	MT_2494	670662	3611157	-0.68674	0.002684	-255.8596
G2.SRR8437784_CS-TJ	G5.SRR8437788_C.Pyrenaica	MT-2414	632048	3529023	-0.696209	0.0024695	-281.9224
G2.SRR8437784_CS-TJ	G5.SRR8437788_C.Pyrenaica	MT-2415	622949	3595179	-0.704633	0.0025496	-276.3702
G2.SRR8437784_CS-TJ	G5.SRR8437790_C.Pyrenaica	MT_2494	675951	3595150	-0.683477	0.0028366	-240.9455
G2.SRR8437784_CS-TJ	G5.SRR8437790_C.Pyrenaica	MT-2414	632637	3518800	-0.69522	0.0024691	-281.5654
G2.SRR8437784_CS-TJ	G5.SRR8437790_C.Pyrenaica	MT-2415	623334	3584660	-0.703738	0.0026041	-270.2474
G2.SRR8437786_CS-KZ	G4.SRR8437789_C.Nubiana	MT_2494	674451	3480731	-0.675369	0.0026331	-256.4915
G2.SRR8437786_CS-KZ	G4.SRR8437789_C.Nubiana	MT-2414	633169	3403279	-0.686274	0.0022995	-298.4498
G2.SRR8437786_CS-KZ	G4.SRR8437789_C.Nubiana	MT-2415	621168	3467219	-0.696131	0.0023935	-290.8444
G2.SRR8437786_CS-KZ	G4.SRR8437792_C.Nubiana	MT_2494	673993	3503623	-0.677331	0.0026794	-252.7963
G2.SRR8437786_CS-KZ	G4.SRR8437792_C.Nubiana	MT-2414	635040	3423521	-0.687062	0.0024945	-275.4319
G2.SRR8437786_CS-KZ	G4.SRR8437792_C.Nubiana	MT-2415	622525	3488195	-0.697121	0.0024905	-279.9154
G2.SRR8437786_CS-KZ	G5.SRR8437787_C.Pyrenaica	MT_2494	673380	3489977	-0.676521	0.0026069	-259.5158
G2.SRR8437786_CS-KZ	G5.SRR8437787_C.Pyrenaica	MT-2414	634955	3413040	-0.686287	0.0024104	-284.7134
G2.SRR8437786_CS-KZ	G5.SRR8437787_C.Pyrenaica	MT-2415	623612	3477908	-0.695912	0.0026317	-264.4385
G2.SRR8437786_CS-KZ	G5.SRR8437788_C.Pyrenaica	MT_2494	674065	3490906	-0.676317	0.0026996	-250.5237
G2.SRR8437786_CS-KZ	G5.SRR8437788_C.Pyrenaica	MT-2414	635668	3413765	-0.686046	0.0024499	-280.0273
G2.SRR8437786_CS-KZ	G5.SRR8437788_C.Pyrenaica	MT-2415	624812	3477367	-0.695376	0.0026111	-266.3111
G2.SRR8437786_CS-KZ	G5.SRR8437790_C.Pyrenaica	MT_2494	679574	3474422	-0.67281	0.0028071	-239.6856
G2.SRR8437786_CS-KZ	G5.SRR8437790_C.Pyrenaica	MT-2414	636223	3402841	-0.684965	0.0023786	-287.9728
G2.SRR8437786_CS-KZ	G5.SRR8437790_C.Pyrenaica	MT-2415	625101	3466809	-0.69447	0.0026414	-262.92
G2.ERR4133453_CS-TJ	G4.ERR4133452_C.Nubiana	MT_2494	654129	3630496	-0.694662	0.0027823	249.6681
G2.ERR4133453_CS-TJ	G4.ERR4133452_C.Nubiana	MT-2414	613705	3569144	-0.706561	0.002364	298.8832
G2.ERR4133453_CS-TJ	G4.ERR4133452_C.Nubiana	MT-2415	601851	3641945	-0.716362	0.0023785	301.1847
G2.SRR5803200_CS-China	G3.SRR5803192_CS-China II	MT_2494	792727	1401014	-0.277283	0.0032918	84.23479
G2.SRR5803200_CS-China	G3.SRR5803192_CS-China II	MT-2414	771914	1381217	-0.282985	0.0031516	89.78979
G2.SRR5803200_CS-China	G3.SRR5803192_CS-China II	MT-2415	777968	1420220	-0.292173	0.0033653	86.82066
G2.SRR5803200_CS-China	G3.SRR5803193_CS-China II	MT_2494	801970	1452285	-0.288483	0.0032236	89.49135
G2.SRR5803200_CS-China	G3.SRR5803193_CS-China II	MT-2414	776932	1428696	-0.2955	0.0030388	97.24316
G2.SRR5803200_CS-China	G3.SRR5803193_CS-China II	MT-2415	782803	1469184	-0.304789	0.0032689	93.24028
G2.SRR5803200_CS-China	G4.ERR4133452_C.Nubiana	MT_2494	623641	3660119	-0.708835	0.0025933	273.338

G2.SRR5803200_CS-China	G4.ERR4133452_C.Nubiana	MT-2414	588769	3600441	-0.718912	0.0021762	330.3593
G2.SRR5803200_CS-China	G4.ERR4133452_C.Nubiana	MT-2415	578436	3677245	-0.728158	0.002247	324.0579
G2.SRR5803204_CS-China	G3.SRR5803192_CS-China II	MT_2494	807519	1402563	-0.269241	0.0035219	76.44863
G2.SRR5803204_CS-China	G3.SRR5803192_CS-China II	MT-2414	783584	1384473	-0.277156	0.00336	82.4856
G2.SRR5803204_CS-China	G3.SRR5803192_CS-China II	MT-2415	790516	1427402	-0.287155	0.0037839	75.88947
G2.SRR5803204_CS-China	G3.SRR5803193_CS-China II	MT_2494	816950	1453992	-0.280519	0.0034497	81.31661
G2.SRR5803204_CS-China	G3.SRR5803193_CS-China II	MT-2414	788614	1432033	-0.289744	0.0032084	90.30915
G2.SRR5803204_CS-China	G3.SRR5803193_CS-China II	MT-2415	795114	1476207	-0.299867	0.0037911	79.09654
G2.SRR5803204_CS-China	G4.ERR4133452_C.Nubiana	MT_2494	636569	3670564	-0.704412	0.0027431	256.7981
G2.SRR5803204_CS-China	G4.ERR4133452_C.Nubiana	MT-2414	598703	3610758	-0.715544	0.0022813	313.6512
G2.SRR5803204_CS-China	G4.ERR4133452_C.Nubiana	MT-2415	586277	3690750	-0.725848	0.0023833	304.5522
G2.SRR8437784_CS-TJ	G3.SRR5803192_CS-China II	MT_2494	834167	1303483	-0.219548	0.0036807	59.64909
G2.SRR8437784_CS-TJ	G3.SRR5803192_CS-China II	MT-2414	808641	1272834	-0.223012	0.0035686	62.49331
G2.SRR8437784_CS-TJ	G3.SRR5803192_CS-China II	MT-2415	818891	1303995	-0.228512	0.0036416	62.75047
G2.SRR8437784_CS-TJ	G3.SRR5803193_CS-China II	MT_2494	845470	1358489	-0.232772	0.0036114	64.45422
G2.SRR8437784_CS-TJ	G3.SRR5803193_CS-China II	MT-2414	815030	1321879	-0.237188	0.00348	68.15829
G2.SRR8437784_CS-TJ	G3.SRR5803193_CS-China II	MT-2415	824367	1354146	-0.243184	0.0035791	67.94529
G2.SRR8437784_CS-TJ	G4.ERR4133452_C.Nubiana	MT_2494	673757	3604779	-0.685053	0.0030632	223.6393
G2.SRR8437784_CS-TJ	G4.ERR4133452_C.Nubiana	MT-2414	632152	3522867	-0.695716	0.0024522	283.7165
G2.SRR8437784_CS-TJ	G4.ERR4133452_C.Nubiana	MT-2415	622009	3588381	-0.704536	0.0024808	283.9913
G2.SRR8437786_CS-KZ	G3.SRR5803192_CS-China II	MT_2494	751341	1101575	-0.189018	0.0032879	57.48864
G2.SRR8437786_CS-KZ	G3.SRR5803192_CS-China II	MT-2414	728813	1076685	-0.192674	0.0032431	59.41007
G2.SRR8437786_CS-KZ	G3.SRR5803192_CS-China II	MT-2415	735048	1102968	-0.200172	0.0033581	59.60877
G2.SRR8437786_CS-KZ	G3.SRR5803193_CS-China II	MT_2494	760750	1153892	-0.205335	0.0032555	63.07337
G2.SRR8437786_CS-KZ	G3.SRR5803193_CS-China II	MT-2414	733213	1123701	-0.210289	0.0030815	68.24268
G2.SRR8437786_CS-KZ	G3.SRR5803193_CS-China II	MT-2415	739627	1152196	-0.21808	0.0032423	67.26127
G2.SRR8437786_CS-KZ	G4.ERR4133452_C.Nubiana	MT_2494	673369	3481012	-0.675827	0.0029773	226.9956
G2.SRR8437786_CS-KZ	G4.ERR4133452_C.Nubiana	MT-2414	633742	3405090	-0.686176	0.0024719	277.5909
G2.SRR8437786_CS-KZ	G4.ERR4133452_C.Nubiana	MT-2415	622794	3468833	-0.695576	0.0026304	264.4343
G2.SRR5803200_CS-China	G2.SRR5803204_CS-China	MT_2494	896232	911198	-0.00828	0.0038987	-2.123842
G2.SRR5803200_CS-China	G2.SRR8437784_CS-TJ	MT_2494	912554	1059447	-0.074489	0.0034014	-21.89937
G2.SRR5803204_CS-China	G2.SRR8437784_CS-TJ	MT_2494	929015	1061193	-0.066414	0.0037071	-17.91554
G2.SRR5803200_CS-China	G2.SRR8437786_CS-KZ	MT_2494	841143	1103747	-0.135023	0.0031638	-42.67688
G2.SRR5803204_CS-China	G2.SRR8437786_CS-KZ	MT_2494	855503	1103669	-0.126669	0.00353	-35.88328
G2.SRR8437784_CS-TJ	G2.SRR8437786_CS-KZ	MT_2494	879009	996325	-0.062557	0.0036059	-17.34841
G2.ERR4133453_CS-TJ	G2.SRR5803200_CS-China	MT-2414	995479	917336	0.0408524	0.0031642	12.91098
G2.ERR4133453_CS-TJ	G2.SRR5803204_CS-China	MT-2414	997681	929314	0.0354786	0.0032835	10.80519
G2.SRR5803200_CS-China	G2.SRR5803204_CS-China	MT-2414	882034	892462	-0.005877	0.0034747	-1.691251
G2.ERR4133453_CS-TJ	G2.SRR8437784_CS-TJ	MT-2414	912665	985365	-0.038303	0.0033551	-11.41616
G2.SRR5803200_CS-China	G2.SRR8437784_CS-TJ	MT-2414	889727	1040003	-0.077874	0.0033115	-23.51606

G2.SRR5803204_CS-China	G2.SRR8437784_CS-TJ	MT-2414	901882	1042371	-0.072259	0.003521	-20.52228
G2.ERR4133453_CS-TJ	G2.SRR8437786_CS-KZ	MT-2414	836781	1024297	-0.100757	0.0030499	-33.03565
G2.SRR5803200_CS-China	G2.SRR8437786_CS-KZ	MT-2414	819094	1083860	-0.139134	0.0031052	-44.80719
G2.SRR5803204_CS-China	G2.SRR8437786_CS-KZ	MT-2414	830095	1085730	-0.133433	0.0032881	-40.58097
G2.SRR8437784_CS-TJ	G2.SRR8437786_CS-KZ	MT-2414	852745	968533	-0.063575	0.0036558	-17.39003
G2.ERR4133453_CS-TJ	G2.SRR5803200_CS-China	MT-2415	1020791	939188	0.0416346	0.0034616	12.02766
G2.ERR4133453_CS-TJ	G2.SRR5803204_CS-China	MT-2415	1025998	950374	0.0382641	0.0037085	10.31803
G2.SRR5803200_CS-China	G2.SRR5803204_CS-China	MT-2415	907848	915231	-0.00405	0.0039545	-1.024078
G2.ERR4133453_CS-TJ	G2.SRR8437784_CS-TJ	MT-2415	928951	1011188	-0.042387	0.0032814	-12.91726
G2.SRR5803200_CS-China	G2.SRR8437784_CS-TJ	MT-2415	906921	1069681	-0.082343	0.003371	-24.42695
G2.SRR5803204_CS-China	G2.SRR8437784_CS-TJ	MT-2415	919189	1075285	-0.078264	0.0038903	-20.118
G2.ERR4133453_CS-TJ	G2.SRR8437786_CS-KZ	MT-2415	852966	1049481	-0.103296	0.0030094	-34.32403
G2.SRR5803200_CS-China	G2.SRR8437786_CS-KZ	MT-2415	834690	1113082	-0.142928	0.003233	-44.20946
G2.SRR5803204_CS-China	G2.SRR8437786_CS-KZ	MT-2415	845494	1117278	-0.13847	0.0036603	-37.83052
G2.SRR8437784_CS-TJ	G2.SRR8437786_CS-KZ	MT-2415	870864	987477	-0.062751	0.003466	-18.10494

Table S4. Score value of D-statistics keeping Himalayan ibex at H3 shows greater affinity between H1 and H3.

H1	H2	H3	nABBA	nBABA	Dstat	SE	Z
G3.SRR5803192_CS-China II	G3.SRR5803193_CS-China II	MT_2494	523814	561339	-0.0345804	0.0026303	-13.14694
G3.SRR5803192_CS-China II	G3.SRR5803193_CS-China II	MT-2414	504182	542873	-0.0369522	0.00263671	-14.0145
G3.SRR5803192_CS-China II	G3.SRR5803193_CS-China II	MT-2415	511727	552416	-0.0382364	0.00265677	-14.39205

Table S5. Comparative assessment of elevation profile within the specific area of interest (India–Tajikistan) between present and paleo DEMs (3 MYA).

Category	Present			Past (3 MYA)		
	Mean spatial distance (upper and lower limit of geo-point #6) (km)	Geometric mean (±SD)	Coefficient of variation	Mean spatial distance (upper and lower limit of geo-point #6) (km)	Geometric mean (±SD)	Coefficient of variation
Valleys with lower elevation gradient	260.16	1943.63 ±73.55	3.78	88.26	1601.37 ±34.72	2.17
Mountain peaks with higher elevation	114.88	3801.25 ±64.81	1.70	226.14	2754.87 ±40.29	1.46

Table S6. Descriptive statistics of elevation profiles within the area of interest between present and paleo DEMs (3 MYA).

N	Present Lower	Lower conf.	Upper conf.	Present higher	Lower conf.	Upper conf.	3 mya lower	Lower conf.	Upper conf.	3 mya higher	Lower conf.	Upper conf.
	**169.00**	**169.00**	**169.00**	**76.00**	**76.00**	**76.00**	**75.00**	**75.00**	**75.00**	**230.00**	**230.00**	**230.00**
Min	1783.54	-	-	3633.35	-	-	1565.12	-	-	2665.46	-	-
Max	2048.94	-	-	3899.78	-	-	1725.46	-	-	2822.48	-	-
Sum	328473.40	326554.90	330321.80	288936.90	287830.30	290074.80	120129.80	119533.00	120691.80	633688.90	632504.30	634903.20
Mean	1943.63	1932.28	1954.57	3801.80	3787.24	3816.77	1601.73	1593.77	1609.22	2755.17	2750.02	2760.45
Std. error	5.66	5.28	6.02	7.43	6.32	8.43	4.01	2.95	4.72	2.66	2.46	2.85
Variance	5409.44	4704.43	6126.55	4200.40	3031.06	5402.69	1205.28	652.50	1674.35	1623.62	1396.55	1867.23
Stand. dev	73.55	68.90	78.60	64.81	56.34	74.87	34.72	27.51	42.30	40.29	37.57	43.44
Median	1935.87	1890.56	1961.97	3804.08	3778.92	3827.66	1589.41	1569.01	1598.36	2765.32	2759.82	2774.61
25 prcntil	1887.70	1882.45	1904.00	3761.50	3745.82	3790.55	1572.11	1565.89	1575.12	2726.58	2721.02	2733.97
75 prcntil	2016.71	2009.12	2027.98	3859.74	3853.11	3873.64	1628.83	1623.91	1640.62	2787.21	2783.51	2791.09
Skew-ness	-0.14	-0.39	0.12	-0.49	-0.93	-0.13	1.24	0.75	2.20	-0.50	-0.70	-0.29
Kurtosis	-1.25	-1.49	-1.03	-0.33	-1.11	0.36	2.00	0.26	5.48	-0.74	-1.11	-0.47
Geom. mean	1942.24	1930.88	1953.17	3801.25	3786.60	3816.24	1601.37	1593.53	1608.77	2754.87	2749.70	2760.15
Coeff. var	3.78	3.54	4.05	1.70	1.48	1.97	2.17	1.73	2.64	1.46	1.36	1.58

Table S7. Key resources table.

Deposited Data	Source
Genome for Himalayan ibex	https://www.ncbi.nlm.nih.gov/sra; detail in Table S6
Genomes for the domestic goat, Tajikistan Siberian ibex, Kazakhstan Siberian ibex, China Siberian ibex, Alpine ibex, Nubian ibex, Iberian ibex, and Bezoar	https://www.ncbi.nlm.nih.gov/sra; detail in Table S6
ANGSD v0.931	https://bitbucket.org/nygcresearch/treemix/wiki/Home
ASTRAL-III v5.7.5	https://github.com/smirarab/ASTRAL
BCFtools v1.4-7-g41827a3	http://samtools.github.io/bcftools/howtos/index.html
Beagle v4.1	https://faculty.washington.edu/browning/beagle/b4_1.html
BWA v0.7.5a-r405	http://bio-bwa.sourceforge.net/
FigTree v1.3.1	http://tree.bio.ed.ac.uk/software/figtree/
GATK v3.7.0	https://software.broadinstitute.org/gatk/
MEGA7	https://megasoftware.net/
MEGA-CC	https://www.megasoftware.net/
MSMC2	https://github.com/stschiff/msmc
MUSCLE-3.8.31	http://www.drive5.com/muscle/
NOVOPlasty2.7.2	https://github.com/ndierckx/NOVOPlasty
Picard v1.56	http://broadinstitute.github.io/picard/
PSMC	https://github.com/lh3/psmc
R	https://www.r-project.org/
RAxML-v8.2.12	https://github.com/stamatak/standard-RAxML
Samtools v1.3.1	http://samtools.sourceforge.net/
TreeMix v1.13	https://bitbucket.org/nygcresearch/treemix/src/master/
VCFtools v0.1.13	https://vcftools.github.io/index.html
FastTree program	http://www.microbesonline.org/fasttree/
WorldClim database	https://worldclim.org/data/index.html
PaleoDEM	https://www.earthbyte.org/paleodem-resource-scotese-and-wright-2018/
R-script for plotting ABBA-BABA or D-statistics result	library(ggplot2)<br>library(RColorBrewer)<br>library(reshape2)<br>d<-read.table("11.IW01-X-Ibex.abbababa.plot.txt",head=T)<br>head(d)<br>orders <- scan(file = "11.IW01-X-Ibex.abbababa.plot-orderID.txt", what = "character")<br>dplot <- ggplot(data = d, aes(x = D,y=H2))<br>dplot <- dplot + geom_pointrange(aes(xmin = D - 3*SE, xmax = D + 3*SE, color= C))<br>geom_point(size=0.1)+scale_color_manual(values = my.colours)<br>dplot + scale_y_discrete(limits=orders)+ geom_vline(aes(xintercept=0),colour="red",line-type="dashed") + xlab("D(C.Sibirica_TJ,H2,C.Sibirica_MT-2494)")
